# Editorial: Immune-checkpoint inhibitors and immunometabolic reprogramming in cancer immunotherapy

**DOI:** 10.3389/fimmu.2025.1760910

**Published:** 2025-12-22

**Authors:** Vijay Kumar, Valentina De Falco

**Affiliations:** 1 Laboratory of Tumor Immunology and Immunotherapy, Department of Surgery, Morehouse School of Medicine, Atlanta, GA, United States; 2 Institute of Endotypes in Oncology, Metabolism and Immunology “Gaetano Salvatore”, Department of Biomedical Sciences, National Research Council (CNR), Naples, Italy

**Keywords:** cancer, immune-checkpoint inhibitor (ICI), immunometabolic reprogramming, immunometabolism, immunotherapy

Tumors and Cancers have always remained a challenge for physicians (both ancient and modern). Surgical removal of the tumor or cancerous tissue is the oldest known medical intervention in oncology (1). With advancements in basic and medical sciences, chemo- and radiotherapies have further advanced therapeutic approaches to treat various cancers. However, these chemo- and radiotherapies come with severe toxicities (2). With advancements in immunological sciences and the establishment of the critical role of the immune system in defense against cancer, immunotherapies emerged (3, 4). These immunological advances revolutionized the fields of cancer biology and oncology, establishing a new branch called cancer/tumor immunology, with associated therapeutics known as cancer immunotherapies (3). Immune-checkpoint inhibitors (ICIs), targeting inhibitory receptors expressed on immune cells to suppress their immunosuppressive actions, are among the most advanced immunotherapies against cancer, and their discoverers (Tasuku Honjo and James Allison) received the 2018 Nobel Prize in Physiology or Medicine (5). Now, several immunosuppressive receptors have been identified and targeted to develop various ICIs to treat different cancers (6). Moreover, immune cell functions, such as anti- and pro-cancer, are governed by their metabolic state, known as immunometabolism (7, 8). Cancer cells, via direct or indirect routes, such as direct cancer and immune cell interactions or through secreted metabolites and factors (cytokines), govern immune cell metabolic reprogramming or immunometabolic reprogramming (IR) that might also be affected by ICIs (7). Therefore, the Research Topic is designed to understand the roles of ICIs and IR in cancer immunotherapies.

This Research Topic provides a comprehensive overview, from clinical meta-analyses of the esophagus, cervix, melanoma, and NSCLC to mechanistic studies on immunometabolism, microbiota, epigenetics, and cardiovascular toxicity, illustrating the integration of immunology and metabolism in precision cancer medicine with ICIs.

The article by Xiong et al. has identified the regulatory role of the NADPH oxidase 4 (NOX4)/MYC axis in murine breast cancer and in immune cell (specifically CD8^+^T cells) infiltration, via dysregulation of breast cancer cell metabolism that supports their growth and proliferation, and PD-L1 overexpression that inhibits the antitumor immune response. Earlier studies have suggested pro- and anti-tumor actions of NOX4 in different cancers, and, given its role in cell metabolism and PD-L1 expression, it would be interesting to understand the link between ICIs and immunometabolic alterations (9, 10). Furthermore, this Research Topic contains two systematic reviews on ICIs by Idibulla et al., and Chen et al., which discuss the efficacy and safety of different ICIs in patients with cervical cancer (CC) and perioperative patients with NSCLC. The meta-analysis by Idibulla et al. has suggested the significant efficacy of ICIs in patients with advanced CC, as indicated by improved overall survival (OS) with a manageable safety profile. On the other hand, a meta-analysis of 8 randomized clinical trials (RCTs) of ICIs in perioperative patients with NSCLC by Chen et al. indicates greater efficacy of ICIs compared with chemotherapy. However, their combination (Toripalimab, an ICI targeting programmed cell death protein 1 (PD-1), and chemotherapy) provides the most significant improvement in event-free survival (EFS). Hence, ICIs (Toripalimab and Nivolumab) are better molecules to fight cancer, and their efficacy increases in conjunction with available chemo- and radiotherapies that can convert cold tumors into hot tumors.

Furthermore, a case report study involving an older woman (69 years old) diagnosed with locally advanced hypopharyngeal carcinoma with cervical esophageal involvement receiving weekly paclitaxel + carboplatin combined with cetuximab (PCC), during which she received pembrolizumab (a PD-1 inhibiting ICI) every 3 weeks for 18 weeks and showed complete response (CR) (Yu et al.). Therefore, the patient was maintained on pembrolizumab only and did not show any sign of tumor recurrence on multiple follow-up examinations, and did not require any surgery or radiotherapy. Thus, patients receiving neoadjuvant immunotherapies to exhibit CR can be maintained on ICIs without cancer remission. The greater efficacy of ICIs in cancer patients who have received neoadjuvant immunotherapy may be explained by the conversion of a cold tumor immune microenvironment (TIME) to a hot one, where ICIs exhibit greater efficacy (11–13). Moreover, Luo et al., using a mouse model of lung cancer and patients with NSCLC, have shown the increased efficacy of ICIs in combination with low-dose radiotherapy (LDR). The present combination suppressed the antioxidant defense mechanism (nuclear factor erythroid-2 related factor 2 (NRF2)/hemeoxygenase-1 (HO-1)/glutathione peroxidase 4 (GPX4) axis) that promoted increased reactive oxygen species (ROS) and free radical generation in response to LDR causing ferroptosis among cancer cells as indicated by the iron (Fe^2+^) accumulation that is critical for converting cold TIME to hot TIME, where ICIs exhibit better efficacy (14). However, ferroptosis of T cells, such as antitumor CD8^+^T cells, supports cold TIME (15, 16). Therefore, further studies are needed to strengthen Luo et al.’s findings in different cancers.

On the other hand, a case report by Wang et al. suggests a possible link between the induction of regenerative hepatic pseudotumor in a 66-year-old man with metastatic NSCLC receiving PD-1/PD-L1 inhibitor (Tislelizumab); otherwise, the side effects associated with ICIs are within a controllable range, which do not impact subsequent surgeries and efficacy. Han et al. have discussed the increased risk of atherosclerosis and other adverse vascular events (AVEs) associated with ICIs in patients with cancer, and ICI discontinuation elevates tumor progression risk and leads to a median treatment delay. However, further articles by Zhang et al., and Zheng et al., have discussed different strategies (selection of cancer patients, combination therapies, and biomarker-guided therapies) to reduce the ICI-associated toxicities, such as immune-related adverse events (irAEs) causing hepato, endocrine, and cardiovascular toxicities, and increase their efficacies to target cancer. Moreover, Zheng et al. have discussed that ICI-induced cardiovascular toxicities can be targeted by IR targeting within immune cells, such as macrophages and T cells, which play critical roles in cardiovascular physiology and associated disease pathologies.

Furthermore, Liu et al., have discussed mechanisms and therapeutic potentials of currently available ICIs targeting different immun-checkpoint proteins/molecules (cytotoxic T lymphocyte-associated protein 4 (CTLA-4), PD-1, T cell immunoglobulin and immune receptor tyrosine inhibitory motif domains (TIGIT), lymphocyte activated gene 3 (LAG-3), V-domain immunoglobulin suppressor of T cell activation (VISTA), T-cell immunoglobulin and mucin domain-3 (TIM-3), signal-regulatory protein alpha (SIRPα), and OX40 or CD134) belonging to different protein families, like immunoglobulin superfamily (IgSF, include PD-1, CTLA4, VISTA, LAG-3, B and T lymphocyte attenuator (BTLA), and CD28), poliovirus receptor (PVR, include TIGIT, CD96, CD226, as well as their ligands CD155 and CD112), T cell/transmembrane, immunoglobulin, and mucin (TIM, include TIM1, 3, and 4 family members), Signal regulatory proteins (SIRP, include SIRPα, SIRPβ1, SIRPγ, SIRPβ2, and SIRPδ family members) and Tumor Necrosis Factor Superfamily (TNSF, include TNFSF-TNFRSF system). This provides the necessary context to interpret new combinatorial designs and understand when local metabolism (lactate, oxygen, amino acids, and iron) influences the gearing of the immune signal by altering immunometabolism. The updated map allows us to move from a “one-size-fits-all” approach to therapeutic constellations tailored to specific immunometabolic phenotypes. For example, Ou et al., in their systematic review and meta-analysis paper, have indicated that elevated neutrophil to lymphocyte ratio (NLR), platelet to lymphocyte ratio (LMR), and derived NLR (dNLR) in patients with melanoma receiving ICIs are associated with poor OS and progression-free survival (PFS). On the other hand, a high lymphocyte-to-monocyte ratio (LMR) is associated with better OS and PFS. Thus, the IR responsible for NLR, PLR, LMR, and dNLR in response to ICIs in patients with melanoma and other cancers is a critical factor to understand during ICI treatment and to validate these findings.

Moreover, environmental factors, such as exposure to ultraviolet radiation (UVR), may also affect ICI efficacy. For example, Zhang et al. utilized the pan-cancer transcriptomic dataset from the Cancer Genome Atlas (TCGA) to understand the association between UVR-related genes and immunosuppression across 30 cancer types, excluding three cancer types comprising primarily of immune cells, such as acute myeloid leukemia (AML), diffuse large B-cell lymphoma, and thymoma (Zhang et al.). The authors found that differentially expressed genes (DEGs) associated with UVR-Sig. are associated with ICI resistance and inhibitory immune cell infiltration in the tumor microenvironment (TME) and pro-tumor pathways (angiogenesis, epithelial-mesenchymal transition (EMT), Hedgehog signaling, IL-2-STAT signaling, IL-6-JAK-STAT signaling, and inflammatory response in tumors with high UVR.Sig expression) in pan-cancer data. Moreover, in their breast cancer study, they observed a strong association between higher Hub-UVR.Sig expression (ENO2 or enolase-2 or gamma enolase, critical for glycolysis and gluconeogenesis, and ATP6V1F, which encodes vacuolar ATPase or V-ATPase, mediates acidification of intracellular organelles) and substantial immune evasion and low immunogenicity in patients with worse OS. Thus, understanding the association between UV exposure, ICI treatment, and associated IR is critical to exploring a linkage between UVR and ICI resistance. Furthermore, Yang et al., and Idibulla et al., have discussed the use, efficacy, and safety of ICIs in gynecological cancers, specifically triple-negative breast cancer (TNBC) and cervical cancer (CC). Yang et al. have updated the information on ongoing clinical trials of ICIs and other immunotherapies against triple-negative breast cancer (TNBC), depending on their subtypes, along with strategies to improve existing immunotherapies. Additionally, another systematic review and meta-analysis by Ren et al. has suggested that patients with esophageal squamous cell carcinoma (ESCC) expressing higher PD-L1 levels exhibit better response to ICIs targeting PD-1/PD-L1 interactions, with this effect further increased in patients of Asian origin, males, smokers, and liver metastasis. Moreover, PD-1/PD-L1 inhibitors (ICIs) in combination with chemotherapies exhibit increased efficacy against ESCC. Interestingly, chemotherapies exhibit chemoimmunomodulation that might involve IR as it governs immune cells’ pro- and anti-cancer function (7, 8, 17, 18). Therefore, understanding immune cell-specific IR in the TME of different cancers, including TNBCs and ESCC, can further increase the efficacy of ICIs, as TME IR has the potential to predict patient response (7, 19). For example, improving CD8^+^T cell bioenergetics or immunometabolism in patients with melanoma restores the tumor’s sensitivity to ICIs (20). Furthermore, patients with metastatic renal cell carcinoma (RCC) who exhibit exceptional responses to ICIs show enriched metabolic pathways and low tertiary lymphoid structure (TLS) signatures, which are associated with improved PFS and OS (21).

Microbiota, metabolism, immunity, and epigenetics are critical mediators of host well-being (22). For example, immune homeostasis is critical for the host’s well-being and requires teamwork among the microbiota, metabolism, epigenetics, circadian clock, endocrine system, and hypothalamus-pituitary (HPA) axis (23). Ren et al. have suggested the role of the microbiota-metabolism-epigenetics-immunity axis in cancer pathogenesis through various aspects: microbiota and metabolism affect the effectiveness of ICIs, epigenetics serves as a bridge between metabolism and immunity, and microbiota-derived metabolites, such as indole-3 propionic acid (IPA), enhance the efficacy of ICIs. Thus, efficacies of ICIs can be impacted by several factors, including TIME, metabolism, epigenetics, and microbiota. Moreover, tumor-associated microbiota or intratumoral microbiota have gained attention recently, which may affect TIME and associated IR and ICI efficacy as well (24, 25). Hence, we must understand the ICI and IR axis to develop novel approaches for cancer immunotherapies.
